# Enterovirus 71 induces dsRNA/PKR-dependent cytoplasmic redistribution of GRP78/BiP to promote viral replication

**DOI:** 10.1038/emi.2016.20

**Published:** 2016-03-23

**Authors:** Jia-Rong Jheng, Shin-Chyang Wang, Chao-Rih Jheng, Jim-Tong Horng

**Affiliations:** 1 Graduate Institute of Biomedical Sciences and Department of Biochemistry and Molecular Biology, College of Medicine, Chang Gung University, Taoyuan 333, Taiwan; 2 Research Center for Emerging Viral Infections, College of Medicine, Chang Gung University, Taoyuan 333, Taiwan; 3 Department of Medical Research, Chang Gung Memorial Hospital, Taoyuan 333, Taiwan; 4 Research Center for Industry of Human Ecology and Graduate Institute of Health Industry Technology, Chang Gung University of Science and Technology, Taoyuan 333, Taiwan

**Keywords:** endoplasmic reticulum stress, enterovirus 71, GRP78/BiP, protein kinase R, unfolded protein response

## Abstract

GRP78/BiP is an endoplasmic reticulum (ER) chaperone protein with the important function of maintaining ER homeostasis, and the overexpression of GRP78/BiP alleviates ER stress. Our previous studies showed that infection with enterovirus 71 (EV71), a (+)RNA picornavirus, induced GRP78/BiP upregulation; however, ectopic GRP78/BiP overexpression in ER downregulates virus replication and viral particle formation. The fact that a virus infection increases GRP78/BiP expression, which is unfavorable for virus replication, is counterintuitive. In this study, we found that the GRP78/BiP protein level was elevated in the cytoplasm instead of in the ER in EV71-infected cells. Cells transfected with polyinosinic–polycytidylic acid, a synthetic analog of replicative double-stranded RNA (dsRNA), but not with viral proteins, also exhibited upregulation and elevation of GRP78/BiP in the cytosol. Our results further demonstrate that EV71 infections induce the dsRNA/protein kinase R-dependent cytosolic accumulation of GRP78/BiP. The overexpression of a GRP78/BiP mutant lacking a KDEL retention signal failed to inhibit both dithiothreitol-induced eIF2α phosphorylation and viral replication in the context of viral protein synthesis and viral titers. These data revealed that EV71 infection might cause upregulation and aberrant redistribution of GRP78/BiP to the cytosol, thereby facilitating virus replication.

## INTRODUCTION

Enterovirus 71 (EV71) belongs to the enterovirus genus of Picornaviridae, which infect mostly infants or children younger than five years and may cause hand, foot and mouth disease.^1^ Neurological complications, including encephalitis and aseptic meningitis, are frequently manifested upon severe infection.^2^ EV71 particles lack an envelope and consist of an icosahedral capsid and a 7.4-kb single positive-stranded RNA. The viral RNA encodes a polyprotein that is subsequently cleaved by viral proteases (2A^pro^, 3C^pro^ and 3CD^pro^) to produce individual viral proteins: structural proteins (VP4, VP2, VP3 and VP1) constitute the capsid shell, whereas nonstructural proteins (2A, 2B, 2C, 2BC, 3A, 3B, 3AB, 3C, 3D and 3CD) are associated with viral replication. In addition, a double-stranded RNA (dsRNA) intermediate produced during virus replication has been shown to trigger antiviral responses or alter cellular processes.^3^ dsRNA-dependent protein kinase R (PKR) is a dsRNA-binding protein that consists of an N-terminal dsRNA-binding domain and a C-terminal kinase domain.^4^ After activation by dsRNA, PKR can phosphorylate eIF2α, which results in the global arrest of protein synthesis. However, some genes that are involved in the integrated stress response (ISR), such as ATF4 and CHOP, are selectively activated under eIF2α phosphorylation to relieve stress or regulate cell death.^5^

The molecular chaperone 78-kDa glucose-regulated protein (GRP78), also referred to as binding immunoglobulin protein (BiP) or heat-shock 70-kDa protein A5 (HSPA5), is expressed primarily in the endoplasmic reticulum (ER).^6^ GRP78/BiP contains an N-terminal ATPase domain, a C-terminal polypeptide-binding domain, and a highly helical C-terminal tail with a KDEL ER retention signal.^7, 8, 9, 10^ The function of GRP78/BiP has previously been shown differ by subcellular localization.^11^ For example, the GRP78/BiP expressed on the cell surface is reported to serve as a receptor for cancer cells to modulate cellular signaling pathways, such as mitogen-activated protein kinase (p38 MAPK), extracellular-signal-regulated kinase 1/2 (ERK1/2), c-Jun N-terminal kinases (JNKs), and phosphoinositide 3-kinase (PI3K).^12, 13^ In addition, a cytosolic GRP78/BiP isoform (GRP78va) may be critical for cell viability.^14^ These findings unveiled a potent antiapoptotic role for GRP78/BiP; therefore, it might be an ideal target for cancer therapy.

Dengue virus infection induces an increase in BiP78/BiP on the cell surface, where it acts as a receptor for virus binding and entry.^15^ In addition, the interaction between hepatitis B virus (HBV) precore protein and GRP78/BiP causes the redistribution of GRP78/BiP from the ER to the cytoplasm, which may account for persistent HBV infection.^16^ GRP78/BiP is a well-known monitor of ER stress and has fundamental roles in the unfolded-protein response (UPR).^17^ Under unstressed conditions, BiP/GRP78 associates with ER stress sensor proteins, for example, inositol-requiring protein 1 (IRE1), protein kinase RNA-activated (PKR)-like ER kinase (PERK) and activating transcription factor 6 (ATF6), to inactivate the sensor proteins. Under stressed conditions, the accumulation of unfolded and misfolded proteins recruits GRP78/BiP, which dissociates sensor proteins from GRP78/BiP and activates the downstream signal transduction cascade to rescue ER homeostasis. An increase in GRP78/BiP in the ER compartment has often been used as evidence of UPR induction. Many viruses manipulate ER stress or UPR to benefit their own replication,^18^ including Japanese encephalitis virus, hepatitis C virus and dengue viruses.^19, 20, 21, 22^ Our previous findings demonstrated that EV71 activated and modulated the UPR for viral replication.^23^ The exogenous expression of GRP78/BiP attenuated EV71-induced ER stress and reduced viral RNA expression and infectious particle formation. Although these results have highlighted that ER stress or the modulated UPR promotes virus replication, the mechanism by which virus subverts GRP78/BiP, whose ectopic overexpression is disadvantageous to the virus, has not yet been identified. Therefore, we hypothesized that GRP78/BiP might lose its function of mitigating ER stress upon EV71 infection and explored the modulation of GRP78/BiP in virus-infected cells.

## MATERIALS AND METHODS

### Cell culture and infection

Human rhabdomyosarcoma (RD) and human embryonic kidney (HEK)293T cells were obtained from American Type Culture Collection (Manassas, VA, USA) and maintained in Dulbecco's modified Eagle's medium (DMEM) (Gibco BRL, Gaithersburg, MD, USA) supplemented with 10% (v/v) fetal bovine serum (FBS) (JRH Biosciences, Lenexa, KS, USA) at 37 °C in a 5% CO_2_-humidified environment. The EV71 (TW/2231/98, genotype C) infectious clone was generously provided by Dr Mei*-*Shang Ho of Academia Sinica, Taiwan. The virus was generated from the infectious clone and propagated in the RD cells, and the virus titer was determined by a plaque assay with RD cells.^24^ UV-inactivated EV71 (UV-EV71) was prepared as described previously.^25^ Viral infection was carried out by preadsorbing EV71 at a multiplicity of infection (MOI) of 10 for 1 h (–1 to 0 h post infection (p.i.)) at 37 °C, and the unbound virus was aspirated and washed with phosphate-buffered saline (PBS). The cells were then cultured in DMEM containing 2% FBS and harvested at the indicated times p.i.

### Reagents and antibodies

Dithiothreitol (DTT), p38 MAPK inhibitor SB203580, Q-VD-OPh, anti-Flag antibody, dimethyl sulfoxide (DMSO) and polyinosinic–polycytidylic acid (poly(I:C)) were purchased from Sigma-Aldrich (St Louis, MO, USA). The JNK inhibitor SP600125 was purchased from BioVision (Milpitas, CA, USA). Staurosporine was purchased from Cell Signaling Technology (Danvers, MA, USA). Mouse anti-PKR antibody was obtained from Santa Cruz Biotechnology (Santa Cruz, CA, USA). Rabbit anti-p-PKR, anti-KDELR, anti-Bax and anti-Calnexin antibodies were purchased from GeneTex (Irvine, CA, USA). Mouse anti-GRP78 and anti-GAPDH antibodies were obtained from BD Transduction (San Diego, CA, USA) and Abnova (Taipei, Taiwan), respectively. Rabbit polyclonal antibodies against the EV71 3A and 2C proteins were prepared as described previously.^26^ The monoclonal antibody against EV71 3D was kindly provided by Dr Shin-Ru Shih of Chang Gung University, Taoyuan.^27^ The anti-mouse IgG (H+L) and anti-rabbit IgG (H+L) antibodies were purchased from Millipore (Billerica, MA, USA). Mouse anti-HA antibody was purchased from Roche (Basel, Switzerland). The Plasma Membrane Protein Extraction Kit (ab65400) was purchased from Abcam (Cambridge, UK).

### Subcellular fractionation

Cells were washed with PBS and resuspended in Bud Buffer (38 mM potassium aspartate, 38 mM potassium gluconate, 20 mM MOPS, 5 mM reduced glutathione, 5 mM sodium carbonate, 2.5 mM magnesium sulfate, pH 7.2) containing protease inhibitors (Roche). All chemicals were purchased from Sigma-Aldrich unless otherwise stated. The samples were homogenized with a cell cracker (HGN Laboratory Equipment, Heidelberg, Germany), and the resulting homogenates were centrifuged at 1000*g* for 10 min at 4 °C. The postnuclear supernatant (PNS) was loaded into the top of a step OptiPrep (Sigma-Aldrich) gradient (10, 20, 30 and 40% discontinuously), followed by centrifugation at 50000*g* for 18 h at 4 °C in a SW 55 Ti rotor (Beckman, Columbia, MD, USA). Fractions (0.3 mL each) were collected from the top of the gradient, and 40 μL of each fraction was analyzed by sodium dodecyl sulfate–polyacrylamide gel electrophoresis (SDS–PAGE) and immunoblotting. For cytosolic/microsomal fractionation, the PNSs were centrifuged at 7000*g* for 10 min at 4 °C. The mitochondrial-rich pellet was discarded, and 300 μg of supernatant was spun at 65 000*g* for 45 min at 4 °C in a Beckman TLA 100.2 rotor to obtain cytosolic supernatant and a microsome-rich pellet. The microsome-rich pellet was dissolved in Bud Buffer.

### Secretion assay

The cells were cultured in DMEM without FBS in a 10-cm culture dish and infected with EV71/2231 for 1 h. Next, they were washed with PBS to remove unbound virus and maintained in DMEM supplemented with 2% FBS for 2 h. The cells were then washed again with PBS and cultured in DMEM only for 6 h. After incubation, the cell culture medium and attached cells were processed separately as described below. The cell culture medium was carefully transferred to 10-kDa Amicon centrifugation filter units (Millipore) and centrifuged at 8000*g* for 45 min at 4 °C. The retentate was resuspended in lysis buffer (1% Triton X-100, 50 mM sodium chloride, 1 mM EDTA, 1 mM EGTA, 20 mM sodium fluoride, 20 mM sodium pyrophosphate, 1 mM sodium orthovanadate in 20 mM Tris-HCl, pH 7.4) containing a protease inhibitor cocktail (Roche) and then collected as the supernatant fraction. The attached cells were washed with PBS, resuspended in lysis buffer, and centrifuged at 17 900*g* for 10 min at 4 °C. The supernatant was collected as the whole-cell lysate.

### Plasmid construction, siRNA and transfection

Full-length GRP78/BiP containing silent mutations in the siRNA target regions was cloned by polymerase chain reaction (PCR) using pcDNA3.1-GRP78FL-myc-His B^+^ as a template^23^ and the following primers: 5′-AAG CTG GCT AGC ATG GAG GAG GAG GAC AAG AAG GAG-3′, 5′-TCT TCG AGT GAC AGC AGA TGA CAA GGG TAC AG-3′, 5′-CTG TAC CCT TGT CAT CTG CTG TCA CTC GAA G-3′ and 5′-GAC GGA TAT CAG CAA CTC ATC TTT TTC TGC TGT ATC CTC-3′. GRP78_ΔKDEL was amplified using a different reverse primer 5′-GAC GGA TAT CAG TTC TGC TGT ATC CTC TTC ACC AGT TGG-3′. Both cDNAs were inserted into AS3W-puro-cFlag with NheI and EcoRV restriction enzymes to generate AS3W-GRP78_WT-cFlag and AS3W-GRP78_ΔKDEL-cFlag constructs. Full-length EV71 2B, 2C, 2BC, 3A, 3AB, 3D and 3CD were amplified from the infectious cDNA clone (TW/2231/98) by PCR and cloned into the NheI and EcoRI sites of AS3W-puro-cFlag. The primers for 2B were 5′-AAG CTG GCT AGC ATG GGC GTG TCT GAT TAC ATT AAA G-3′ and 5′-ATC GAT GAA TTC GTC TGC TTC TGA GCC ATC G-3′. The primers for 2C were 5′-AAG CTG GCT AGC ATG AGT GCC TCT TGG TTA AAG-3′ and 5′-CGA CTG AAT TCG TTT GGA AAA GAG CTT CAA TG-3′. The primers for 2BC were 5′-AAG CTG GCT AGC ATG GGC GTG TCT GAT TAC ATT AAA G-3′ and 5′-CGA CTG AAT TCG TTT GGA AAA GAG CTT CAA TG-3′. The primers for 3A were 5′-GCA TGG CTA GCA TGG GAC CCC CTA AAT TTA G-3′ and 5′-ATC GAT GAA TTC GTT TGA AAA CCG GCG AAC AAC-3′. The primers for 3AB were 5′-GCA TGG CTA GCA TGG GAC CCC CTA AAT TTA G-3′ and 5′-ATC GAT GAA TTC GTC TGC ACA GTT GCC GTG C-3′. The primers for 3D were 5′-AAG CTG GCT AGC ATG GGT GAG ATC CAA TGG ATG-3′ and 5′-CGA CTG AAT TCG TAA ACA ATT CGA GCC AAT TTC-3′. The primers for 3CD were 5′-GAT TAG CTA GCA TGG GGC CGA GCT TGG ACTT C-3′ and 5′-CGA CTG AAT TCG TAA ACA ATT CGA GCC AAT TTC-3′. Plasmids expressing 2A^pro^ and 3C^pro^ were cloned into pcDNA3.1-mycHis(−).^28^ Scrambled siRNA (medium GC of Stealth negative control duplex), siRNA against EIF2AK2 (PKR): 5′-UUU ACU UCA CGC UCC GCC UUC UCG U-3′ and siRNA against GRP78/BiP: 5′-UAC CCU UGU CUU CAG CUG UCA CUC G-3′ were purchased from Invitrogen (Carlsbad, CA, USA). The siRNA and constructs were transfected into designated cells using Lipofectamine 2000 (Invitrogen) as described by the manufacturer. The pKH3-KDELR plasmid expressing human KDEL receptor was kindly shared by Dr Guanghui Wang.^29^

### RNA extraction and quantitative PCR analysis

Total RNA was extracted from cells using TRIzol (Invitrogen). After DNase I treatment, 1 μg of total RNA was reverse-transcribed using the M-MLV reverse transcriptase system (Invitrogen). Quantitative PCR was performed using the StepOnePlus Real-Time PCR system (Applied Biosystems, Foster City, CA, USA) with the following specific primer pairs: BiP forward primer 5′-CTC AAC ATG GAT CTG TTC CG-3′ and reverse primer 5′-CCA GTT GCT GAA TCT TTG GA-3′ GAPDH forward primer 5′-TGC ACC ACC AAC TGC TTA GC-3′ and reverse primer 5′-GGC ATG GAC TGT GGT CAT GAG-3′. The target genes were then amplified under the following conditions: 50 °C for 2 min, 95 °C for 10 min, 50 cycles at 95 °C for 15 s and 60 °C for 1 min. To quantify the changes in target gene expression, the comparative Ct (ΔΔCt) method was used to calculate relative fold changes normalized to the GAPDH control. Data are expressed as the mean±s.d. and were analyzed with two-tailed Student's *t*-tests. *P*<0.05 was considered significant.

### SDS–PAGE and western blotting assay

The protein concentration was measured with a protein assay kit (Bio-Rad, Richmond, CA, USA). Equal amounts of cellular protein were analyzed by SDS–PAGE and transferred to polyvinylidene fluoride membranes. The membranes were immunoblotted with specific primary antibodies followed by horseradish peroxidase (HRP)-conjugated secondary antibodies and developed with Immobilon Western HRP Chemiluminescence Substrate (Millipore).

### GRP78/BiP rescue experiments

RD cells were transfected with either scrambled or GRP78 siRNA for three days. Then, AS3W-puro-cFlag (empty vector), AS3W-GRP78_WT-cFlag or AS3W-GRP78_ΔKDEL-cFlag construct was transfected into cells for another 24 h. After infecting with EV71 at an MOI of 10 for 6 h, the cell lysates were prepared for western blotting. The viral progeny inside the cells and in the culture medium were pooled for a plaque assay.^24^ Data are expressed as the mean±s.d. and were analyzed with two-tailed Student's *t*-tests. *** indicates *P*<0.001.

## RESULTS

### EV71 infection induces redistribution of GRP78/BiP

**Figure 1:**
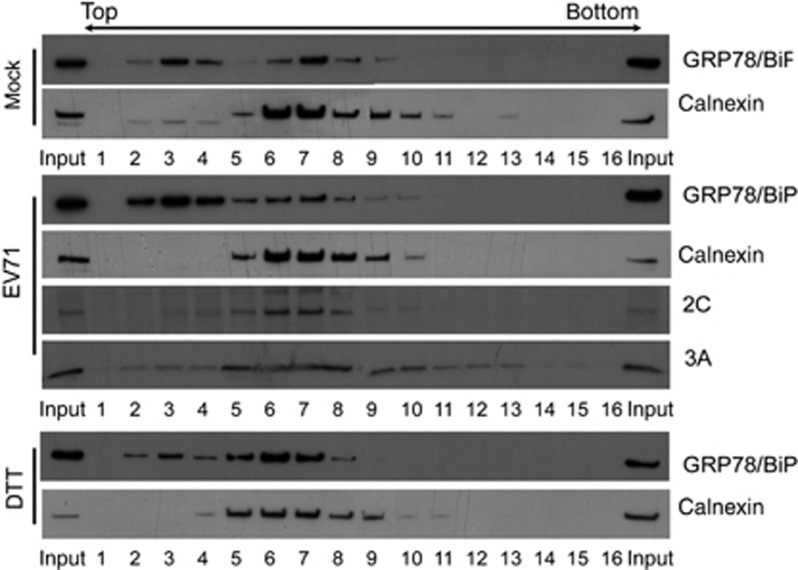
Subcellular fractionation profiles of GRP78/BiP in mock-infected, EV71-infected or DTT-treated RD cells. The PNSs from mock-infected, EV71-infected (MOI of 10, 6 h p.i.), 2.5 mM DTT-treated (6 h) cells were fractionated using OptiPrep gradients. The fractions were collected from the top and subjected to SDS–PAGE and subsequent western blotting using specific antibodies against GRP78/BiP, calnexin, and viral proteins 3A and 2C. The results are representative of three independent experiments.

**Figure 2:**
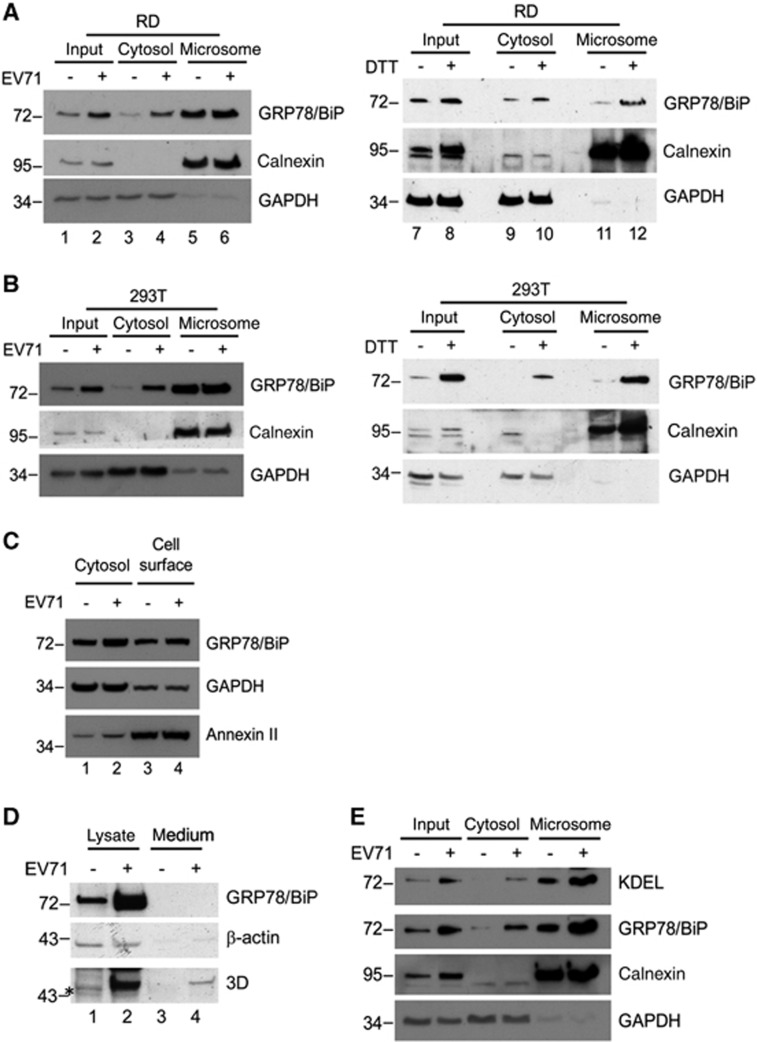
EV71 infection induced an intracellular redistribution of GRP78/BiP. (**A**, **B** and **E**) Cytoplasmic/microsomal localization of GRP78/BiP during viral infection. Homogenates extracted from mock, EV71-infected (MOI of 10, 6 h p.i.) or DTT-treated (2.5 mM, 6 h) RD (A and E) or HEK293T cells (B) were subjected to cytosolic/microsomal fractionations. After centrifugation, fractionates were resolved on SDS–PAGE followed by western blotting with antibodies against the indicated proteins. (**C**) Cell surface distribution of GRP78/BiP in EV71-infected RD cells. RD cells were infected with EV71 at an MOI of 10, and the membrane proteins were prepared using a Plasma Membrane Protein Extraction Kit at 6 h p.i. Annexin II and GAPDH served as markers for the plasma membrane and cytosol fractions, respectively. (**D**) Immunoblot analysis of cell lysates and culture medium obtained from mock-infected or EV71-infected cultures. Thirty micrograms of lysates or Amicon filter unit-concentrated supernatant was subjected to SDS–PAGE and western blotting. Calnexin, GAPDH and β-actin were used as markers for microsomes, cytoplasm and internal controls, respectively. The results are representative of three independent experiments. An asterisk marks nonspecific bands.

We previously showed that EV71 infection induced and modified the UPR.^23^ The ER stress marker GRP78/BiP was upregulated at the protein level in response to viral infection. Because GRP78/BiP can target different subcellular compartments in response to stimuli,^11^ we investigated the cellular localization of GRP78/BiP following EV71 infection by fractionating PNS obtained from mock-infected, EV71-infected or DTT (ER stress inducer)-treated RD cells on a step OptiPrep gradient (Figure 1). Two main pools of GRP78/BiP were detected in the mock control gradient: one coincided with calnexin (fractions 6–9, designated as heavier fractions), an ER transmembrane protein and the other was present in the lighter fractions (fractions 2–4). We found that in DTT-treated cells, GRP78/BiP mostly cofractionated with calnexin in the heavier fractions (fractions 5–8). However, upon virus infection, the majority of GRP78/BiP was found in the lighter fractions (fractions 2–4), whereas viral nonstructural proteins 2C and 3A strongly cosedimented with calnexin (fractions 6–8). We determined the differential subcellular GRP78/BiP distribution in EV71-infected and DTT-treated RD cells (Figure 1). We further confirmed the results with a different subcellular fractionation approach in which cytosol- and microsome-rich fractions were collected by differential centrifugation and analyzed by western blotting. Antibodies against calnexin and GAPDH were used to confirm the fractionation process. As expected, calnexin was expressed in the microsomal fraction, whereas GAPDH was exclusively found in the cytosol Figure 2A). The total protein expression of GRP78/BiP was upregulated in EV71-infected RD cells (lane 1 versus 2, Figure 2A), but its levels were elevated in the cytosol (lane 3 versus 4) and almost unchanged in the microsomal fractions (lanes 5 and 6, Figure 2A). Conversely, GRP78/BiP was upregulated by DTT, and more GRP78/BiP expression was observed in the microsome-rich fraction (lanes 11 and 12, Figure 2A). The same phenomenon was observed in HEK293T cells (Figure 2B), implying that the aberrant redistribution of GRP78/BiP to the cytosol was independent of cell type. Because a fraction of the GRP78/BiP cellular pool can be secreted from cells upon viral infection^30^ or anchored to the cell surface in some cancer cells,^11^ we next examined the ability of EV71 infection to result in the secretion of GRP78/BiP or cell surface localization in addition to its effect on cytoplasmic redistribution. To detect cell surface GRP78/BiP, plasma membrane proteins from RD cells mock-infected or infected with EV71 (MOI of 10) were collected and extracted 6 h p.i. The extracts were resolved using SDS–PAGE and then analyzed with a western blot. As shown in Figure 2C, virus infection did not alter the cell surface expression level of GRP78/BiP compared with the mock-infected control (lanes 3 and 4, Figure 2C). Annexin II was used as a marker and loading control for membrane proteins. To detect secreted GRP78/BiP, RD cells were maintained in DMEM without FBS and infected with EV71 2231 (MOI of 10) for 1 h. The cells were then washed with PBS and cultured in DMEM supplemented with 2% FBS for 2 h, followed by culturing in DMEM without FBS for another 6 h. The cell culture medium and cell lysates were collected as described in the secretion assay above. The secretion of GRP78/BiP did not differ between mock- and virus-infected cells (lanes 3 and 4, Figure 2D), although GRP78/BiP was upregulated by EV71 (lanes 1 and 2, Figure 2D). The same results were obtained in virus-infected HEK293T cells and SF268 cells (a human neuroblastoma cell line) (data not shown). In addition, previous studies have shown that GRP78/BiP devoid of C-terminal KDEL motif is secreted.^31^ Using the cytosolic/microsomal fractionation assay, we detected both the KDEL recognition (motif) and GRP78/BiP in the cytosolic fraction (Figure 2E). These results further confirmed that EV71 infection did not trigger GRP78/BiP secretion. We concluded that EV71 virus infection induces the intracellular redistribution of GRP78/BiP from the ER to cytoplasm.

### Cytoplasmic elevation of GRP78/BiP does not result from virus-induced KDEL receptor diminution

**Figure 3:**
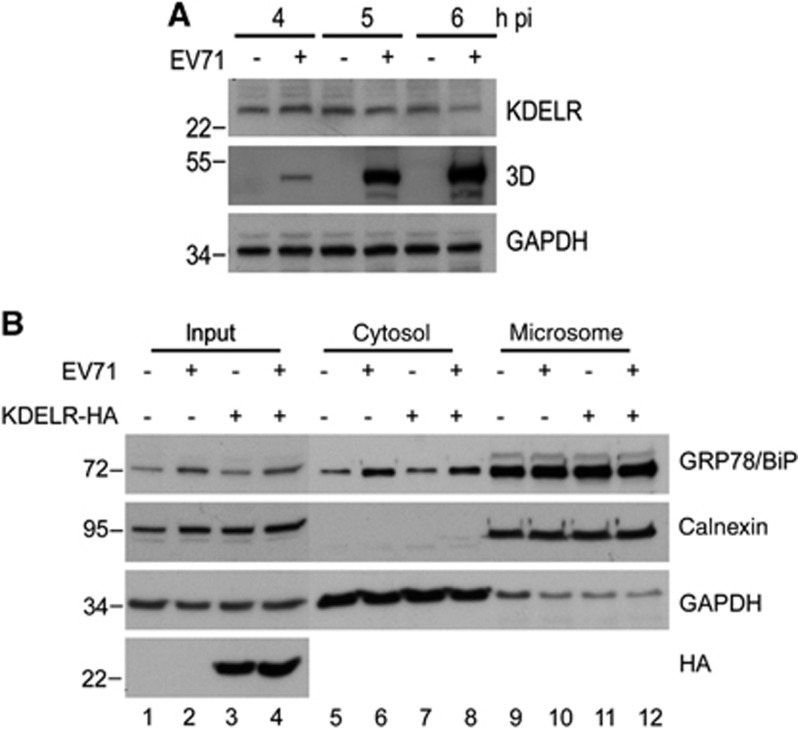
Redistribution of GRP78/BiP was independent of the expression of KDEL receptor. (**A**) RD cells were infected with EV71 (MOI of 10), followed by whole-cell lysate preparation for western blot analysis of the expression levels of KDELR. The viral protein expression is indicated by the levels of viral protein 3D. (**B**) RD cells were transfected with KDELR-HA or vector control for 24 h and then infected with EV71 (MOI of 10). Cell homogenates were collected at 6 h p.i. and subjected to cytosolic/microsomal fractionations and western blot analysis for the distribution of GRP78/BiP. The results are representative of three independent experiments. The expression of KDELR-HA was detected with anti-HA antibodies (bottom panel).

The KDEL receptor (KDELR) is primarily located in the Golgi complex and the ER-Golgi intermediate compartment. It recognizes and retrieves KDEL-containing proteins from the Golgi complex to transport them to the ER.^31, 32^ Thus, we examined the effect of EV71 infection on KDELR expression and its association with the redistribution of GRP78/BiP. We infected RD cells with EV71 and collected cell lysates at the indicated time points p.i. to monitor the expression level of KDELR. The 3D intensity of viral protein became detectable starting at 4 h p.i. and increased in a time-dependent manner until 6 h p.i. However, decreased KDELR levels were detected in EV71-infected cells relative to mock-infected controls after 5 h p.i. (Figure 3A). We further examined the role of KDELR in virus-induced GRP78/BiP redistribution using cytosolic/microsomal fractionation assay in cells overexpressing KDELR. KDELR tagged with HA (KDELR-HA) was transfected into RD cells, and its expression levels were similar in mock- and virus-infected cells, as monitored by western blotting (lanes 3 and 4 of bottom panel, Figure 3B). However, the overexpression of KDELR did not affect virus-induced GRP78/BiP redistribution into the cytosol (lanes 5–12, Figure 3B), indicating that the virus-induced KDELR decreases are not associated with the redistribution of GRP78/BiP. This finding also suggests that the reduction of KDELR upon viral infection might be a result of virus-induced translational attenuation.

### dsRNA is required for the upregulation and redistribution of GRP78/BiP

**Figure 4:**
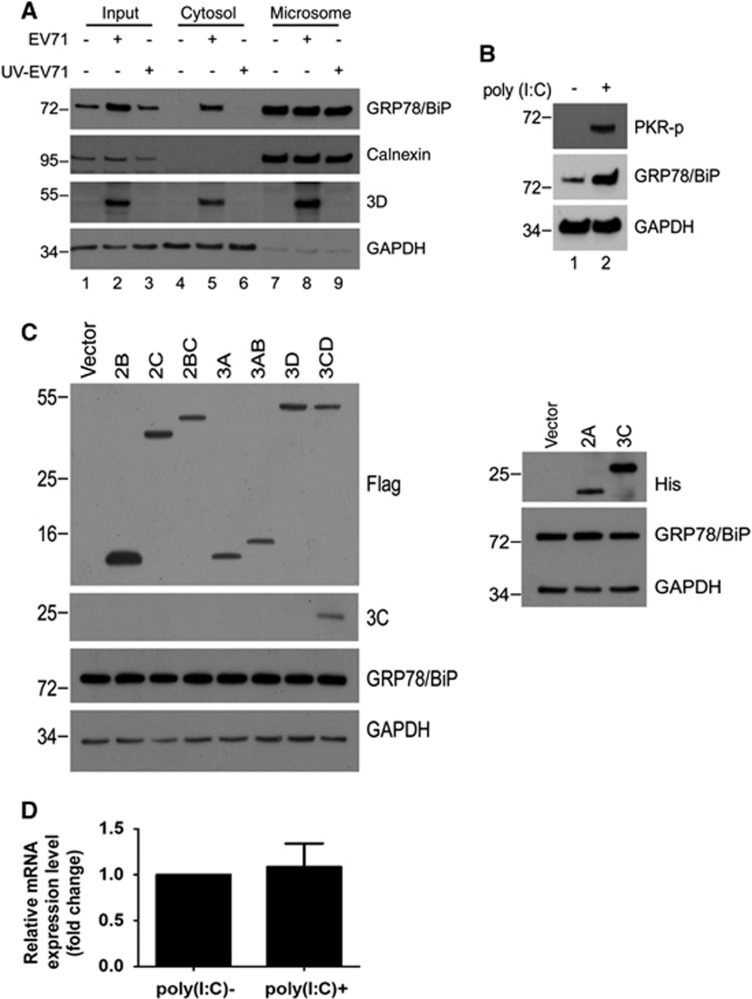
dsRNA, but not viral proteins, induced the upregulation of GRP78/BiP. (**A**) Immunoblot analysis of GRP78/BiP in cytosolic/microsomal fractionations from mock-infected, EV-infected (MOI of 10, 6 h p.i.) or UV-EV71-infected RD cells. The results are representative of three independent experiments. (**B** and **C**) HEK293T cells were transfected with 10 μg/mlof dsRNA analogue poly(I:C) for 18 h (**B**) or Flag or His fusion constructs of individual viral proteins for 24 h (C). The success of poly(I:C) transfection was assessed based on the phosphorylation of PKR (**B**). Whole-cell lysates were analyzed for the viral protein and GRP78/BiP expression levels by western blotting. The expression levels of individual viral proteins were monitored with anti-Flag or anti-His antibodies as indicated (**C**). The results are representative of three independent experiments. (**D**) Expression level of GRP78/BiP mRNA in poly(I:C) transfection. RD cells were transfected with poly(I:C) for 18 h, and the GRP78/BiP mRNA was detected using quantitative real-time RT-PCR. The results are expressed as the means±s.d. (*n*=3). The difference was not significant.

**Figure 5:**
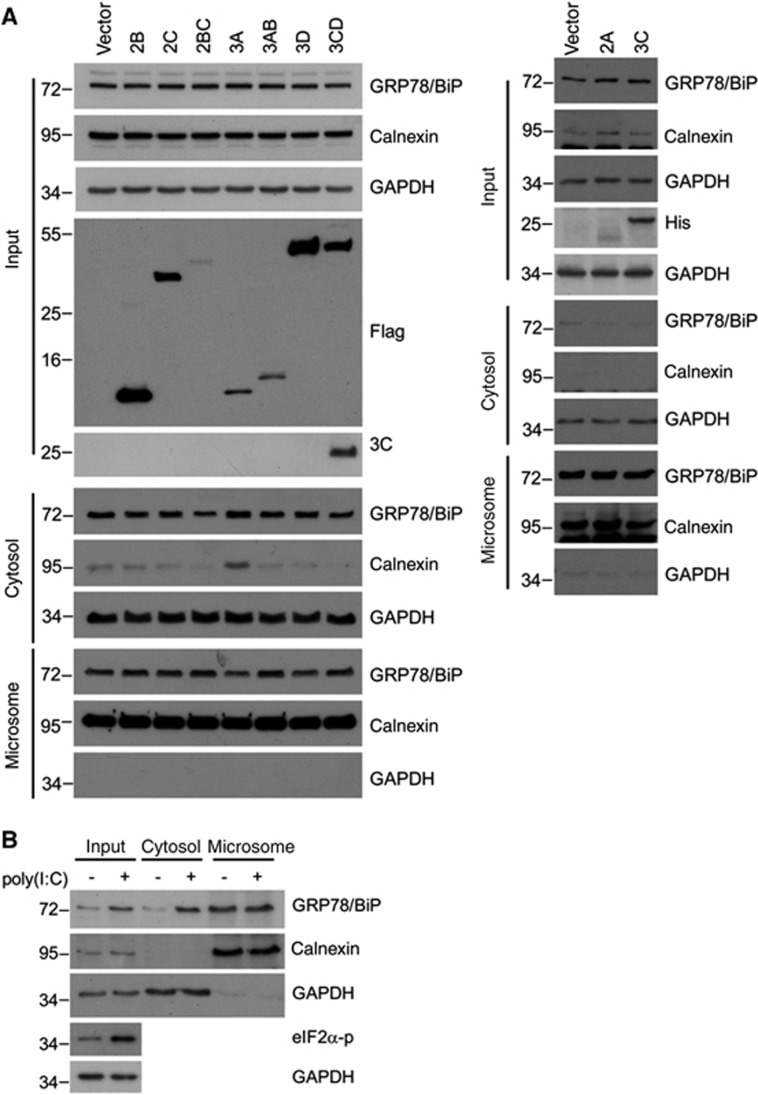
dsRNA, but not viral proteins, is responsible for the redistribution of GRP78/BiP. Immunoblot analysis of GRP78/BiP in cytosolic and microsomal fractions from cells transfected with viral proteins (**A**) or poly(I:C) (**B**). These results are representative of three independent experiments.

We previously showed that the EV71-induced UPR depended on viral replication in host cells because replication-incompetent EV71 due to UV-inactivation no longer exhibited UPR-induction activity.^23^ To explore the necessity of viral replication for GRP78/BiP redistribution, we infected RD cells with mock- or UV-irradiated EV71 (UV-EV71), and subjected the resultant cell homogenates to subcellular fractionation. In contrast to mock-treated EV71, UV-EV71 did not increase the cytosolic GRP78/BiP level (lanes 5 and 6, Figure 4A), indicating that viral replication is necessary for GRP78/BiP redistribution. Next, we explored the ability of viral proteins or replicative viral RNA to upregulate GRP78/BiP and increase its cytoplasmic accumulation. HEK293T cells transfected with individual nonstructural proteins or with poly(I:C), a mimic viral replication intermediate,^33^ were lysed, and the lysates were subjected to western blotting. The results showed dsRNA induced not only the phosphorylation of its downstream target PKR but also resulted in substantial GRP78/BiP expression (Figure 4B). Conversely, the expression of individual viral proteins did not result in elevated levels of GRP78/BiP, although the viral proteins were efficiently expressed (Figure 4C). The quantitative PCR data showed that transfection with poly(I:C) did not increase the mRNA level of GRP78/BiP (Figure 4D), which corroborated the effect of EV71 infection^23^ and indicated that GRP78/BiP is posttranscriptionally regulated regulation by dsRNA. We further determined whether the cytosolic accumulation of GRP78/BiP in response to virus infection was due to dsRNA or the expression of viral proteins. No individual protein changed the expression level of cytosolic GRP78/BiP (Figure 5A), and only in poly(I:C)-transfected cells exhibited increases in cytosolic GRP78/BiP (Figure 5B), indicating a role for dsRNA in the redistribution of GRP78/BiP. Therefore, dsRNA, not the viral protein, is responsible for GRP78/BiP upregulation and cytosolic redistribution.

### PKR mediates virus-induced elevation of cytosolic GRP78/BiP

**Figure 6:**
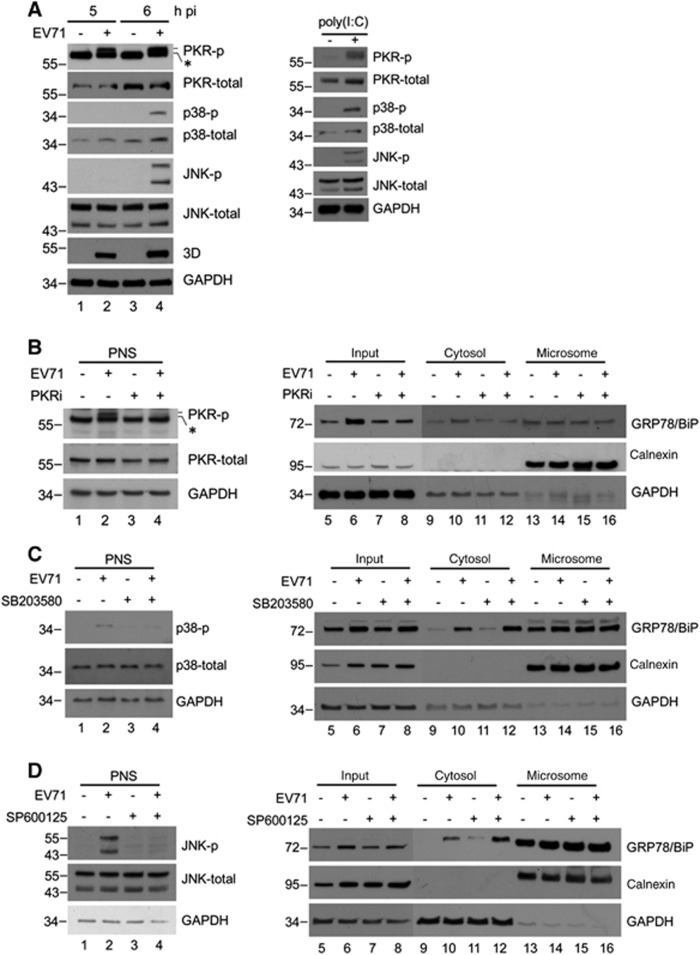
dsRNA induced a PKR-dependent cytosolic elevation of GRP78/BiP on EV71 infection. (**A**) EV71 infection and poly(I:C) transfection led to the phosphorylation of PKR, p38 MAPK and JNK. RD cells were infected with EV71 (MOI of 10, 5 and 6 h, respectively) or transfected with poly(I:C) for 18 h. Cells were lysed for western blotting with antibodies against the indicated proteins. Antibodies against total PKR, total p38 MAPK, total JNK and GAPDH were used to monitor loading. An asterisk marks a nonspecific band. (**B**–**D**) RD cells were infected with EV71 (MOI of 10) or mock-infected and subsequently treated with DMSO, SP600125 (20 μM), SB203580 (20 μM) or PKRi (10 μM) at 3 h p.i. The PNS was collected at 6 h p.i. to examine the levels of p-JNK, p-p38 MAPK and p-PKR by western blotting (left) or subjected to cytosolic/microsomal fractionations to analyze the distribution of GRP78/BiP with a western blot (right). An asterisk marks nonspecific bands. These results are representative of three independent experiments.

Previous reports indicated that PKR, p38 MAPK, and JNK may be activated by dsRNA or virus infection.^34, 35, 36^ Therefore, we confirmed the phosphorylation of PKR, p38 MAPK and JNK in poly(I:C)-treated or virus-infected RD cells (Figure 6A). The results showed that the activation of all these kinases was detectable in both virus-infected and poly(I:C)-treated cells, and their expression levels were similar to those of the mock-infected controls (Figure 6A), indicating that the changes in active kinase levels were not caused by an increase in the expression of total kinases. We next attempted to identify the kinase affects the cytosolic redistribution of GRP78/BiP. We treated cells with inhibitors of PKR (PKRi), p38 MAPK (SB203580) and JNK (SP600125) after virus infection, and the resultant cell homogenates were subjected to subcellular fractionation (Figures 6B–D). The inhibitors effectively abolished the virus-induced phosphorylation in the PNS (lanes 1–4, Figures 6B–D). It is possible that P38 MAPK and JNK did not cause the redistribution because the levels of virus-induced cytosolic GRP78/BiP in SB203580- or SP600125-treated cells were similar to those in control DMSO-treated cells (lanes 6 and 8, Figures 6C and D). However, virus-induced cytosolic GRP78/BiP increases were not observed in cells treated with PKRi (lanes 6 and 8, Figure 6B), indicating that the inhibition of PKR, but not p38 MAPK or JNK, impaired the accumulation of cytosolic GRP78/BiP in response to EV71 infection.

### ISR and apoptosis does not cause the upregulation of cytosolic GRP78/BiP

**Figure 7:**
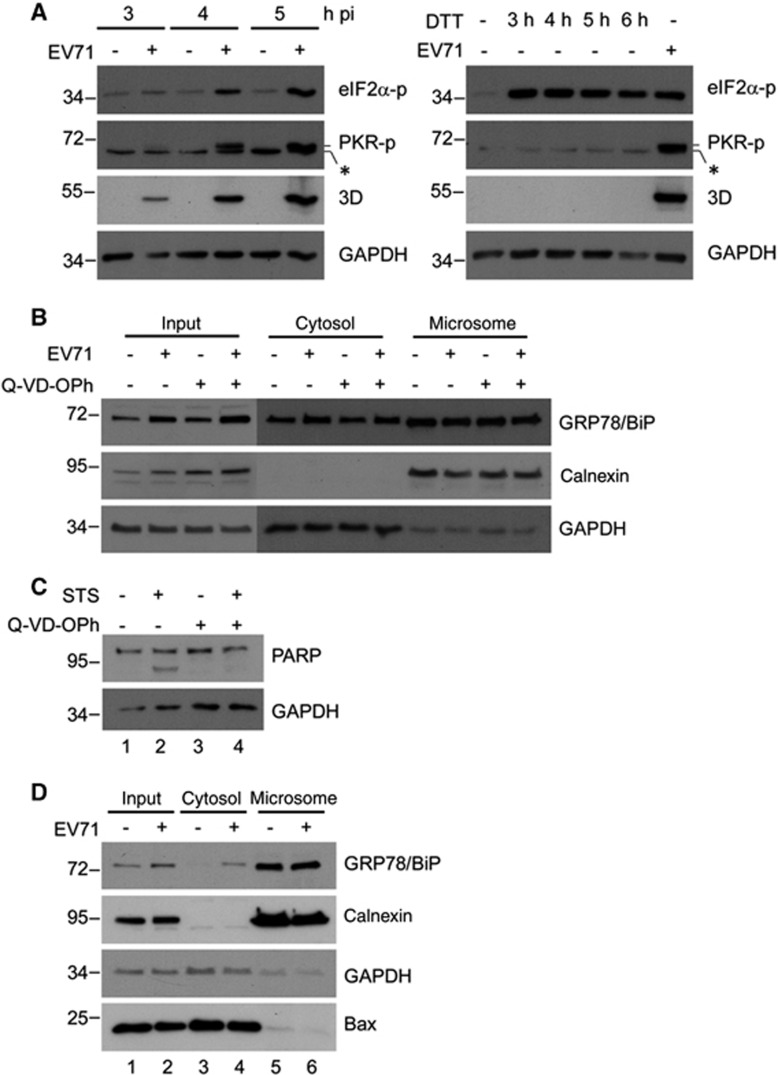
EV71-induced cytosolic elevation of GRP78/BiP did not occur via ISR or apoptosis. (**A**) Time-course study of p-PKR and p-eIF2α in EV71-infected (MOI of 10, left) or DTT-treated (2.5 mM, right) RD cells. An asterisk marks a nonspecific band. (**B**) RD cells were infected with or without EV71 (MOI of 10) and subsequently treated with DMSO or Q-VD-OPh (20 μM) at 0 h p.i. Cell homogenates were collected at 6 h p.i. and subjected to cytosolic/microsomal fractionations and a western blot analysis of the distribution of GRP78/BiP. (**C**) Inhibition of staurosporine-induced PARP-cleavage by Q-VD-OPh. RD cells were treated with 100 nM of staurosporine (STS) in the presence or absence of Q-VD-OPh (20 μM) for 4 h. The cell lysates were collected and subjected to a western blot analysis. (**D**) Relocalization of Bax during viral infection. Cell homogenates extracted from mock- or EV71-infected (MOI of 10, 6 h p.i.) RD cells were subjected to cytosolic/microsomal fractionations. After centrifugation, fractionates were resolved on the SDS–PAGE followed by western blotting with antibodies against the indicated proteins. The results are representative of three independent experiments.

Notably, although both virus infection and DTT treatment induced eIF2α phosphorylation (Figure 7A), DTT treatment did not increase the level of cytosolic GRP78/BiP (Figures 2A and B), ruling out the involvement of ISR in the redistribution of GRP78/BiP. In addition, PKR phosphorylation was observed in cells infected with EV71, but not in cells treated with DTT (Figure 7A), supporting that virus-induced PKR phosphorylation plays a role in GRP78/BiP modulation. Together, these results suggest that PKR is required for the virus-induced increase in cytosolic GRP78/BiP. Because recent studies have shown that PKR mediates apoptosis,^37^ we examined whether the accumulation of cytosolic GRP78/BiP occurs concomitantly with virus-induced apoptosis. To this end, DMSO or Q-VD-OPh, a broad caspase inhibitor, was added to EV71-infected and uninfected cells, and the resultant cell homogenates were subjected to subcellular fractionation. Treatment with Q-VD-OPh did not affect the EV71-induced accumulation of cytosolic GRP78/BiP (Figure 7B). Because caspase activity was difficult to detect by a western blot analysis 6 h p.i., the inhibition efficiency of apoptosis by Q-VD-OPh was demonstrated in a parallel experiment, in which staurosporine-induced poly-(ADP-ribose) polymerase (PARP) cleavage was reduced in response to Q-VD-OPh treatment (Figure 7C). Furthermore, because the ER stress-induced translocation of Bax protein to the ER membrane has been shown to be critical for the release of ER luminal proteins by increasing membrane permeability during ER stress-induced apoptosis,^38, 39^ we next investigated the localization of Bax protein upon EV71 infection. The results of the cytosolic/microsomal fractionation assays showed that virus infection did not alter the subcellular localization of Bax (lanes 5 and 6 of bottom panel, Figure 7D). Together, these results revealed that the upregulation of cytosolic GRP78/BiP is not apparently related to virus (PKR)-induced apoptosis.

### Sustained ER stress caused by the EV71-induced redistribution of GRP78/BiP is advantageous to the virus

**Figure 8:**
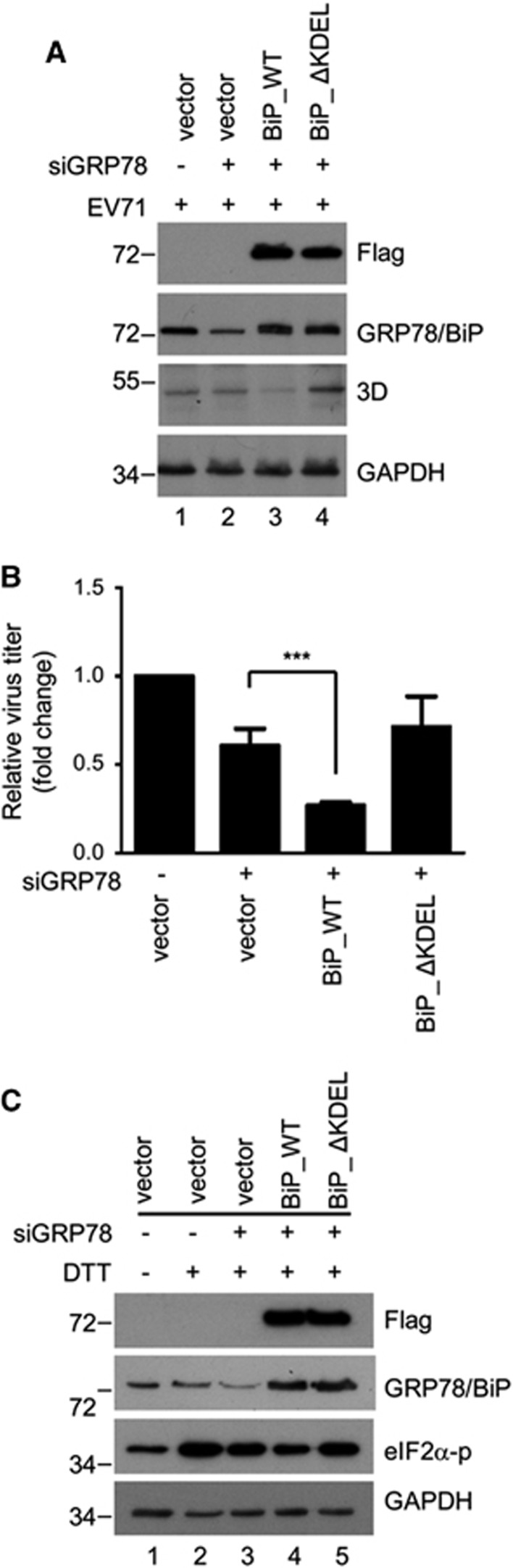
EV71-induced redistribution of GRP78/BiP conferred prolonged ER stress and was advantageous to virus infection. (**A–C**) RNAi rescue experiments were performed by transfecting GRP78/BiP-knockdown RD cells with siRNA-resistant BiP_WT or BiP_ΔKDEL. The cells were then infected with EV71 (MOI of 10, 6 h p.i.). The viral protein expression, as indicated by the 3D level, was assessed by a western blot analysis (A), and the virus titers were determined by plaque assays (B). The plaque assay results are expressed as the means±s.d. (*n*=3). ***P<0.001 compared with the re-expression of vector alone. (C) The cells were treated with 2.5 mM DTT for 4 h, and the cell lysates were then collected to examine the levels of p-eIF2α with a western blotting analysis. These results are representative of three independent experiments.

To explore the effect of GRP78/BiP redistribution on viral protein expression and viral particle production, siRNA-resistant wild-type (GRP78/BiP_WT) or C-terminal KDEL-deleted mutant GRP78/BiP (GRP78/BiP_ΔKDEL) devoid of the ER retention signal KDEL was expressed in RD cells in which endogenous GRP78/BiP was knocked down with specific siRNA. After infection with EV71, the expression level of viral protein and the virus titer were assessed by western blotting and a plaque assay, respectively. Both GRP78/BiP_WT and GRP78/BiP_ΔKDEL were effectively and equally expressed in endogenous GRP78/BiP-silenced RD cells (lanes 3 and 4, Figure 8A). Compared with the vector control, the re-expression of GRP78/BiP_WT suppressed viral protein expression, as indicated by viral 3D protein and viral particle formation, but viral protein expression was not significantly affected in GRP78/BiP_ΔKDEL re-expressing cells (Figures 8A and B). Because ER stress is beneficial for virus replication,^23^ we hypothesized that GRP78/BiP was arrested in the cytosol, leading to sustained ER stress upon virus infection. To verify this hypothesis, the level of DTT-induced eIF2α phosphorylation in GRP78/BiP_WT or GRP78/BiP_ΔKDEL re-expressing cells was investigated. As expected, the DTT-induced eIF2α phosphorylation level decreased in response to transfection with GRP78/BiP_WT, but this phosphorylation was not affected in GRP78/BiP_ΔKDEL re-expressing cells (lanes 4 and 5, Figure 8C). Taken together, these results indicate that EV71 infection induces permanent ER stress by redistributing GRP78/BiP to facilitate virus infection.

## DISCUSSION

The ER is a crucial organelle that supports picornavirus replication. It is considered a source of precursor membranes for viral replication complex formation.^40, 41, 42^ In addition, our previous study showed that viral 2C protein interacts with the host ER transmembrane protein reticulon 3 and is required for EV71 replication.^26^ Recently, the ER-derived autophagosome-like vesicle that participates in nonlytic viral spread and virus infection has emerged as a topic in picornavirus studies.^43, 44^ Therefore, learning how viruses modulate the ER membrane or ER proteins may provide new insights for the antiviral research.

We previously demonstrated that EV71 infection increased the GRP78/BiP protein levels in RD cells.^23^ Paradoxically, the ectopic expression of GRP78/BiP not only alleviated ER stress but also inhibited viral replication. In this study, results from the OptiPrep gradient showed that EV71 infection altered the distribution of GRP78/BiP (Figure 1). We further performed secretory assays, plasma membrane protein extraction assays, and cytosolic/microsomal fractionation assays to show that EV71 infection increased the level GRP78/BiP in the cytosol but not in the ER (Figure 2). We suggest that this cytosolic increase in GRP78/BiP facilitates viral replication.

The expression of GRP78/BiP is conventionally regulated at both the transcriptional and posttranscriptional levels. The GRP78/BiP promoter is primarily activated by ER stress-response elements (ERSEs) under stressed conditions, and these elements share a consensus sequence: CCAAT(N_9_)CCACG.^45^ In addition, the ATF4-binding site has also been reported to localize to an ATF/CRE sequence upstream of the ERSEs and contribute to GRP78/BiP induction.^46^ The posttranscriptional regulation of GRP78/BiP is mediated by the activation of internal ribosome entry sequence (IRES) in the 5′ untranslated region of GRP78/BiP mRNA^47^ or by an increase in protein stability.^48, 49^

Some viruses enforce the expression of GRP78/BiP. For example, human cytomegalovirus induces GRP78/BiP by increasing transcription and translation.^50^ Hepatitis C virus E2 envelope protein activates the GRP78/BiP promoter.^51^ We previously reported that EV71 infection induces GRP78/BiP in an ATF6-independent manner, but this induction does not depend on the activation of ERSE promoter of GRP78/BiP.^23^ The quantitative PCR results demonstrated that dsRNA posttranscriptionally upregulates GRP78/BiP expression (Figures 4B and D). We postulate that EV71 infection activates GRP78/BiP IRES and leads to GRP78/BiP accumulation. In support of this hypothesis, previous studies showed that dsRNA guides translational repression,^52, 53^ and IRES-mediated translation is enhanced when cap-dependent protein synthesis is decreased.^54^ Furthermore, poliovirus, a member of the Picornaviridae family, reportedly increases the translation of GRP78/BiP mRNA in a cap-independent manner.^55^ PI3K/Akt has also been shown to promote GRP78/BiP stability under conditions of ER stress.^48^ Because EV71 infection induces PI3K/Akt activation,^25^ we cannot exclude the possibility that EV71 might enhance GRP78/BiP stability by activating PI3K/Akt. In addition, as posttranscriptional regulators, microRNAs also play a role in GRP78/BiP regulation. miR-181 has been reported to directly target GRP78/BiP mRNA and repress its expression by suppressing translation. In our current study, we found that EV71 infection leads to a decrease in miR-181 (unpublished data). Thus, the mechanism by which EV71 infection upregulates GRP78/BiP expression will be an interesting topic for future studies.

Some mechanisms have been reported for the redistribution of GRP78/BiP to the cytosol: (1) GRP78/BiP is relocated to the cytoplasm via ER-associated degradation (ERAD);^16^ (2) Bcl-2 family proteins, including Bax and Bak, modulate ER membrane permeability to luminal proteins during ER stress-induced apoptosis;^38^ and (3) cytosolic GRP78va that lacks the ER signaling peptide is generated by the alternative splicing of mRNA.^14^ In our previous reports, we demonstrated that EV71 upregulated the XBP1 mRNA level, but the IRE1-mediated XBP1 splicing and transcription of genes that encode ERAD associated proteins was not activated,^23, 28^ indicating that EV71 infection does not activate ERAD. In this study, we found that virus infection did not change the electrophoretic mobility of the GRP78/BiP and the subcellular localization of Bax (Figure 7D), implying a not-yet-identified mechanism of GRP78/BiP regulation upon EV71 infection. Interestingly, the data obtained from cells transfected with dsRNA or individual viral protein indicated that only dsRNA was involved in the cytosolic elevation of GRP78/BiP (Figure 5). Thus, the dsRNA-mediated signaling pathway was hypothesized to cause the redistribution of GRP78/BiP. We examined three dsRNA signaling pathways, and only the inhibition of PKR activity significantly attenuated the virus-induced upregulation of cytosolic GRP78/BiP (Figure 6A). Therefore, we suggest that the dsRNA-PKR signaling axis is involved in the EV71-induced redistribution of GRP78/BiP.

The investigation of the effect of GRP78/BiP redistribution on viral protein expression and viral particle production indicates that permanent ER stress caused by the redistribution of GRP78/BiP is beneficial to EV71 (Figure 8), which is consistent with our previous study.^23^ However, because other roles have been reported for cytosolic GRP78/BiP, GRP78/BiP redistribution may facilitate EV71 replication in many ways. GRP78/BiP reportedly protects cells against ER stress-induced apoptosis by forming a complex with caspase-7 and caspase-12 to prevent the release of caspase-12 from the ER.^56^ In addition, GRP78va can interact with P58IPK and reduce its protein level, which in turn specifically enhances PERK signaling and protects cells from ER stress-induced cell death.^14^ Accordingly, growing evidence shows that viruses may suppress apoptosis for the efficient production of mature virus, which maximizes viral infectivity;^57, 58^ therefore, the cytosolic relocalization of GRP78/BiP may promote viral infectivity by suppressing apoptosis. Furthermore, an interaction between HBV precore protein and cytosolic GRP78/BiP may enhance viral persistence.^16^ In our study, viral RNA-dependent RNA polymerase 3D cofractionated with GRP78/BiP both in the lighter fractions of the OptiPrep gradient (data not shown) and the cytosolic part of cytosolic/microsomal fractionation (Figure 4A) upon EV71 infection. Thus, cytosolic GRP78/BiP may play a role in facilitating viral protein folding or enhancing virus replication. In summary, our findings suggest that PKR-mediated GRP78/BiP redistribution is proviral for EV71. This should be further explored as a potential antiviral target for the inhibition of EV71.
